# The economic burden of anxiety and depression in Indonesia: evidence from a cross-sectional web panel survey

**DOI:** 10.3389/fpubh.2025.1667726

**Published:** 2025-10-15

**Authors:** Karen Arulsamy, Elmeida Effendy, Sarah Mardhiyah, Mustafa M. Amin, M. Surya Husada, Vita Camellia, Anne-Claire Stona, Eric Andrew Finkelstein

**Affiliations:** ^1^Lien Centre for Palliative Care, Duke-NUS Medical School, Singapore, Singapore; ^2^Department of Psychiatry, Universitas Sumatera Utara, Medan, Indonesia; ^3^SingHealth-Duke Global Health Institute, Duke-NUS Medical School, Singapore, Singapore

**Keywords:** Indonesia, cost-of-illness, depression, anxiety, economic burden

## Abstract

**Background:**

Mental health conditions are the second leading cause of disability in Indonesia, accounting for 13 percent of total years lived with disability. However, little is known about their broader economic impact. This study estimates the economic burden of anxiety and depression in adults, including healthcare costs and productivity losses, using a low-cost web panel approach that can be replicated in countries lacking data.

**Methods:**

A cross-sectional online survey was conducted with 5,828 Indonesian adults via a web panel. Participants completed the Patient Health Questionnaire-4 (PHQ-4) for themselves and household members, providing data on 16,096 individuals. Participants who screened positive for anxiety and/or depression symptoms based on the PHQ-4 (N = 438) were then asked about their healthcare utilization, days missed from work, and reduced productivity due to these symptoms. These responses were monetized and extrapolated based on the prevalence rate and population counts to generate per person and total annual costs.

**Results:**

Overall, 14.7 percent reported symptoms consistent with anxiety or depression, yet over 60 percent were never formally diagnosed, highlighting a large diagnosis gap. Direct healthcare costs averaged IDR 2,111,020 per person annually. Employees reported 34 missed workdays per year and were 51 percent less productive while working. Indirect costs via absenteeism and presenteeism averaged IDR 5,178,312 and IDR 11,021,700 per person. The total annual economic burden was IDR 463,811.33 billion (USD $29.22 billion), or 2.1 percent of Indonesia’s GDP, with labor market productivity losses accounting for 88.5 percent of the total.

**Conclusion:**

Anxiety and depression impose substantial health and economic costs in Indonesia. Low-cost, evidence-based interventions—particularly workplace-focused programs—could generate significant health and economic benefits.

## 1 Background

Mental health conditions are a leading cause of disability globally, with anxiety and depression contributing to nearly half of the global disease burden (1). The high disease burden of these conditions imposes tremendous costs to the economy in terms of higher healthcare utilization and increased absenteeism and presenteeism in the workplace. Lost productivity due to anxiety and depression alone is estimated to cost the global economy US$1 trillion per year and is expected to reach US$16 trillion by 2030 (2, 3). In the US, the overall economic burden of diagnosed depression accounts for 1.6 percent of GDP (4). The costs of all mental health conditions, with anxiety and depression being the most significant, are estimated to account for approximately 4–5 percent of GDP in Europe and the UK (5–7). When only the disability component is taken into account, mental health disorders account for 25.3 and 33.5 percent of all years lived with a disability in low to middle income countries (LMICs) respectively (8). Scarce resources constrain mental healthcare provision in LMICs. On average, less than 2 percent of health budgets are allocated to mental healthcare in LMICs compared to 4 percent in high income countries (9). Moreover, a high treatment gap in LMICs result in a larger burden of undiagnosed cases (10). Evidence on the overall economic burden of anxiety and depression in LMICs, particularly in Southeast Asia, is limited.

Our study addresses this gap by quantifying the economic burden of diagnosed and undiagnosed anxiety and depression in terms of mental healthcare utilization and labor productivity losses among adults in Indonesia. Mental health conditions are the second leading cause of disability in the country, accounting for 13 percent of total years lived with disability (11). The Basic Health Survey 2018 (Laporan Nasional Riskesdas) estimated that 6.1 per cent of Indonesians over 15 years old were diagnosed with depression based on the Mini International Neuropsychiatric Interview (12). Several studies have also documented prevalence rates for depression based on the nationally representative Indonesian Family Life Survey (IFLS) using the 10-item version of the Center for Epidemiologic Studies Depression Scale (CES-D) (13). Based on data from the most recent wave of IFLS (2014–2015), Peltzer and Pengpid reported a prevalence of moderate and severe depression of 21.8 percent among adults (14). Focusing on younger adults, another study based on IFLS documented a depression prevalence rate of 27.9 percent (15). However, the 2018 Basic Health Survey and IFLS do not provide publicly available data on anxiety rates. Further, while the Basic Health Survey collects information on mental healthcare utilization, it does not capture labor productivity losses associated with depression (12). Absenteeism and presenteeism (i.e., reduced productivity while working) typically account for a large share of the economic burden of anxiety and depression given their significant effects on concentration, fatigue, and motivation (16).

To estimate the economic burden of anxiety and depression in Indonesia, we employ a web panel approach. Web panels have increased in popularity in recent years as an economical solution to provide timely and credible data to guide resource allocation and policy (17–19). In this study, prevalence estimates are based on the validated Patient Health Questionnaire-4 (PHQ-4) which assesses core symptoms of depression and anxiety (20). Our economic burden estimates comprise three key measures: healthcare expenditures based on monetizing the value of self-reported mental healthcare utilization; absenteeism based on the market value of self-reported days missed from work; and presenteeism defined as the monetized value of self-reported reductions in productivity while working due to anxiety and depression symptoms. We also present results on the diagnosis gap (i.e., individuals who report symptoms based on the PHQ-4 but have not been formally diagnosed), differences in prevalences rates by key demographic characteristics, and the distribution of productivity losses by symptom severity. This approach is easily replicable in countries without current data and provides a model that can be adapted to different contexts.

## 2 Methods

### 2.1 Participants

We fielded a cross-sectional online survey from August 14 to November 18 2024 to Indonesian citizens aged at least 18 years old who are part of an online platform hosted by TGM Research.

Respondents were first asked to complete the Patient Health Questionnaire-4 (PHQ-4) for themselves and on behalf of all other adult household members, based on their experiences over the past 2 weeks. The PHQ-4 consists of the 2-item Patient Health Questionnaire-2 (PHQ-2) and the Generalized Anxiety Disorder-2 (GAD-2). The PHQ-2 and GAD-2 have high sensitivity (83 percent and 88 percent respectively) and specificity (90 percent and 82 percent respectively) in detecting symptoms of anxiety and depression (20). Respondents were asked “Over the last 2 weeks, how often have you been bothered by the following problems?” and responded to four items (“Feeling down, depressed or hopeless”; “Little interest or pleasure in doing things”; “Feeling nervous, anxious or on edge; and “Not being able to stop or control worrying”). These items are scored on a scale with the response options “not at all,” “several days,” “more than half the days,” and “nearly every day,” which are scored as 0, 1, 2, and 3, respectively. This results in a score that ranges from 0 to 6 for each subscale – a score of ≥ 3 on either subscale is the validated threshold for detecting probable cases of anxiety and depression (20, 21). Due to its brevity and ease of administration, the PHQ-4 is commonly used as a screener for anxiety and depression including in clinical settings (22). If a main respondent reported symptoms of anxiety and depression as indicated by this threshold, they were then asked to complete a longer survey, which took about 20–25 min to complete (20). This portion of the survey contains detailed questions on the main respondent’s healthcare utilization and labor market productivity to estimate the economic burden associated with these conditions. We also fielded the Patient Health Questionnaire-9 (PHQ-9) and Generalized Anxiety Disorder-7 (GAD-7) to assess symptom severity (23, 24). This approach provides us with three key measures of interest as detailed below.

### 2.2 Measures

#### 2.2.1 Prevalence rates of anxiety and depression

As respondents complete the PHQ-4 for themselves and for other adult household members, we can estimate the overall prevalence rates of anxiety and depression among adults. Based on this approach, we obtained PHQ-4 data for 16,096 individuals provided by 5,828 main respondents. Prevalence rates are estimated by dividing the number of individuals who scored 3 or more on the PHQ-2 or GAD-2 by the total number of individuals across all households in the sample. Respondents are also asked questions on whether they or other adult household members had been previously diagnosed with anxiety and depression by a physician. By comparing responses to these questions and the PHQ-4, we established the diagnosis gap in the sample. We also assess statistical differences in the prevalence of anxiety and depression by gender, age groups, and geographical region using tests of proportions.

#### 2.2.2 Healthcare utilization

The survey included questions on the use of medication, number of outpatient visits to healthcare providers, and occurrences of serious medical events (hospitalizations and emergency department visits) for anxiety and depression symptoms. These questions are modeled based on the Medical Expenditures Panel Survey (MEPS) (25). Respondents are asked whether they are currently taking any medications to manage their symptoms and subsequently the frequency (as needed or daily) and duration (less than 1 month, 1 to 6 months, and more than 6 months) of consumption. Healthcare providers included non-specialist providers, psychiatrists, and psychologists and we assessed the number of visits in the past 3 months. We multiplied number of visits by four to obtain estimated number of annual visits. Respondents were also asked questions on whether they visited an emergency department or were hospitalized in the past 12 months due to symptoms of anxiety and depression and subsequently the number of visits and nights spent in hospital, respectively.

To estimate the costs of mental healthcare utilization associated with anxiety and depression, unit costs were applied to each type of service based on case-based tariffs obtained from the Regulation of the Ministry of Health in Indonesia (Permenkes Number 3/ 2023) (26). For our analyses, we take a conservative approach and use the midpoint of the relevant tariffs. Although we gathered data on the number of nights an individual was hospitalized, we did not collect information on the number of hospitalization events. In Indonesia, hospitalization costs tend to remain consistent regardless of the length of stay. Therefore, for those who reported any hospitalization, we conservatively assumed one hospitalization event per year to estimate associated costs. The unit costs used are provided in Supplementary material 1. To obtain per person healthcare cost estimates, we averaged costs across all respondents in the sample including those who report no healthcare utilization. This approach accounts for the fact that not all individuals with anxiety and depression seek treatment.

Total healthcare cost estimates are calculated by multiplying estimated adult citizen population counts by the estimated overall prevalence rate for anxiety and depression and the average per person cost estimates. In terms of population size, we focus on those aged 15–64 based on the most recently released census data in 2020 and exclude foreign citizens, resulting in 172,310,323 individuals (27).

#### 2.2.3 Labor market productivity

Labor market productivity losses associated with anxiety and depression, in terms of absenteeism and presenteeism costs, are estimated based on the human capital approach and elicited via the Workplace Productivity and Impairment Questionnaire: Specific Health Problem V2 fielded to employed respondents (28, 29). Absenteeism was captured by asking respondents to state the number of hours missed from work due to anxiety and depression symptoms in the past week. Presenteeism was captured by asking respondents the degree to which their symptoms affected productivity while working on a scale of 0–10 with 0 being “no symptoms and/or symptoms had no effect on my work” and 10 being “symptoms completely prevented me from working” (29). Each respondent’s ‘absenteeism’ response is multiplied by 48 (number of weeks in a work year) to estimate total hours lost annually and monetized by multiplying by an average hourly wage. Hourly wages are calculated by dividing the average monthly income in Indonesia (IDR 3, 040, 719) by 160, the typical number of working hours per month (30). To calculate presenteeism costs, we first calculate weekly hours missed due to presenteeism by multiplying total hours worked in the past 7 days as reported by respondents by the presenteeism score/10. Hours missed are then annualized and monetized following the same approach for absenteeism. Total costs are calculated by multiplying average person costs with Indonesian adult working population counts and the estimated prevalence rate.

### 2.3 Statistical analyses

We primarily used descriptive analyses to estimate prevalence rates, healthcare utilization, labor productivity, and associated costs, following approaches used in similar studies (17, 18). Significant differences in prevalence rates by sociodemographic groups are assessed using a two-sample z-test for proportions. Significant differences in the distribution of labor market costs by severity of anxiety and depression as captured by the Patient Health Questionnaire-9 (PHQ-9) and Generalized Anxiety Disorder-7 (GAD-7) are assessed using a two-sample z-test for means (23, 24). To assess these differences, we first generated binary indicators for each category of the categorical demographic variables, designating one category as the reference group.

We applied winsorization at the 95th percentile to all continuous healthcare utilization and labor productivity variables to limit the influence of outliers while minimizing sample loss. The 95th percentile was selected as a conservative threshold. This approach involves replacing values larger than the 95th percentile with values at the 95th percentile. Per capita and total cost estimates without winsorization are reported in the Supplementary material 2 (31). Given the high variance in the per capita estimates and results showing that symptoms often co-occur, we combine burden estimates for depression and anxiety symptoms. All costs are reported in IDR and USD.

We requested TGM Research to ensure broad representativeness in terms of gender, age groups, and income breakdowns for the first part of the survey, in which respondents completed the PHQ-4 for themselves and other household members. For the second part of the survey, covering healthcare utilization and labor productivity, no sampling method was specified. Participation in this section depended on the underlying prevalence of anxiety and depression, whether respondents passed the attention check, and their willingness to complete the longer questionnaire. Two pilot tests with over 50 respondents were conducted to refine the survey before full fieldwork. Due to use of copyright material, the survey instrument is available upon request.

### 2.4 Ethics statement

Institutional Review Board (IRB) approval was obtained from the relevant institutions (NUS-IRB-2024-281/500/KEPK/USU/2024). Formal written informed consent was obtained from participants following NUS and USU IRB guidelines.

## 3 Results

### 3.1 Prevalence of anxiety and depression

Table 1 shows the overall sample characteristics including the prevalence rates of anxiety and depression and diagnosis gap. Among 16,096 individuals, 14.7 percent had symptoms consistent with anxiety and/or depression. Among those with symptoms, a majority of respondents had symptoms of anxiety only (5.2 percent) followed by comorbid anxiety and depression (5.5 percent), and depression only (4.0 percent). The extent of comorbidity between anxiety and depression is relatively high with nearly 30 percent having symptoms of both conditions. Importantly, among those with symptoms based on the PHQ-4, 61.4 percent had not previously been diagnosed with anxiety or depression by a physician, indicating a potentially large diagnosis and treatment gap.

**Table 1:** Overall sample characteristics.

Sample characteristics	Proportions (%)/Means
Female (%)	54.5
Age	38.2 (13.8)
Number of adults in the household	3.4 (1.5)
Region (%)	
Bali	1.4
Java	74.0
Kalimantan	3.9
Maluku Islands	0.3
Lesser Sunda Islands	0.7
Western New Guinea	0.0
Papua	0.3
Sulawesi	4.7
Sumatra	14.7
Prevalence rates (%)	
Anxiety	5.2
Depression	4.0
Both anxiety and depression	5.5
Anxiety and/or depression	**14.7**
Diagnosis gap (%)	**61.4**
Number of observations	16,096

This table summarizes the characteristics of the overall sample for which PHQ-4 data were available. The prevalence of anxiety and/or depression was defined as the proportion of individuals who scored ≥3 on either the anxiety or depression subscale of the PHQ-4. Standard deviations provided in parentheses for continuous variables.

In Table 2, we assess whether there are significant differences in prevalence rates of anxiety and/or depression by sex, age, and region. There is no gender gap in prevalence rates as observed in other countries, consistent with IFLS data (14). Respondents aged 36 to 59 years old are significantly less likely to exhibit anxiety and depression symptoms and those aged at least 60 years old are more likely to have symptoms compared to respondents aged 18 to 35 years old. Older individuals report the highest rate of anxiety and depression. In terms of region, respondents in Lesser Sunda Islands and Sulawesi report significantly higher rates of anxiety and depression than individuals in Java.

**Table 2:** Prevalence rates of anxiety and/or depression by sex, age, and region.

Sample characteristics	Prevalence rate of anxiety and/or depression (%)	Test of differences
Sex		
Female	15.0	0.007 (0.006)
Male	14.3	Base
Age		
18–35	15.9	Base
36–59	12.5	−0.034 (0.006) ***
60 years old and above	18.5	0.026 (0.010) **
Region		
Java	13.6	Base
Bali	14.2	−0.005 (0.024)
Kalimantan	15.9	0.017 (0.015)
Maluku Islands	18.2	0.040 (0.048)
Lesser Sunda Islands	24.4	0.102 (0.033) **
Western New Guinea	0	-
Papua	22.6	0.085 (0.049)
Sulawesi	17.0	0.029 (0.013) *
Sumatra	15.7	0.016 (0.008)
Number of observations	16,096	

This table summarizes prevalence rates of anxiety and/or depression by key demographic characteristics. The prevalence of anxiety and/or depression was defined as the proportion of individuals who scored ≥3 on either the anxiety or depression subscale of the PHQ-4. Column 3 presents differences compared to the base group with standard errors provided in parentheses. *, **, *** indicates significance at the 5, 1 and 0.1 percent levels, respectively.

In the following sections, we focus on the sample of main respondents (N = 483) who reported symptoms of anxiety or depression based on the PHQ-4 and filled out the longer survey containing questions on healthcare utilization and labor market productivity. We excluded respondents who failed the attention check question (If offered the choice, would you prefer to receive IDR 100,000 today or IDR 80,000 1 year from now?) and those who initially reported symptoms on the PHQ-4 but later reported no symptoms on the PHQ-9 or GAD-7. This was done to minimize the impact of participant inattention on our results.

### 3.2 Main respondent characteristics in the sample with anxiety and/or depression

Table 3 summarizes the characteristics of the main respondents. Most of the respondents are female, reside in Java, have a tertiary qualification, are single, and earn more than IDR 5,999,999 per month in employment income. Compared to data from the IFLS and the Department of Statistics (Badan Pusat Statistik) in Indonesia, respondents in our sample have higher educational attainment and employment income (30, 32). Among unemployed respondents, 19.5 percent reported that they were unemployed due to their mental health symptoms.

**Table 3:** Main respondent characteristics in the sample with anxiety and/or depression.

Sample characteristics	Proportions (%)/ Means
Female (%)	58.6
Age	28.8 (8.7)
Number of adults in the household	3.0 (1.4)
Region (%)	
Bali	0.8
Java	72.1
Kalimantan	3.9
Maluku Islands	0.2
Lesser Sunda Islands	1.2
Western New Guinea	0.0
Papua	0.8
Sulawesi	3.7
Sumatra	17.2
Education – Tertiary qualification (%)	54.7
Marital Status – Single (%)	56.7
Unemployed (%)	23.8
Unemployed because of anxiety/depression symptoms (%)	19.5
Monthly employment income (%)	
Less than IDR 1,000,000	8.4
IDR 1,000,000 – IDR 1,999,999	14.7
IDR 2,000,000 – IDR 2,999,999	12.0
IDR 3,000,000 – IDR 3,999,999	14.4
IDR 4,000,000 – IDR 4,999,999	9.8
IDR 5,000,000 – IDR 5,999,999	11.7
More than IDR 5,999,999	29.1
Number of observations	483

This table summarizes the characteristics of main respondents who completed the longer survey on healthcare utilization and labor productivity. Inclusion required a score of ≥3 on either the anxiety or depression subscale of the PHQ-4, passing the attention check, and completing the full survey. Standard deviations provided in parentheses for continuous variables.

### 3.3 Mental healthcare utilization

Table 4 provides a detailed breakdown of mental healthcare utilization in the sample. Approximately 40 percent visited a healthcare provider, either in person, online, or tele-based, in the past 3 months for their anxiety and depression symptoms. Among these respondents, 85.7 percent consulted a non-specialist provider, and 66 percent consulted a psychiatrist or psychologist. On average, respondents reported at least two visits to a healthcare provider in the past 3 months. 28 percent of respondents reported currently taking prescription medications for their anxiety and depression symptoms. Most respondents take these medications as needed and have been taking them for more than a month. A smaller proportion (24.4 percent) reported serious medical events in the past 12 months. 15.1 percent visited the emergency department, 8.7 percent visited the emergency department and were subsequently hospitalized, and 15.7 percent were hospitalized directly. Respondents reported at least 2 visits per serious medical event in the past year.

**Table 4:** Healthcare utilization of main respondents in the sample with anxiety and/or depression.

Healthcare utilization	Proportion (%)	Mean visits
Healthcare consultations		
Any healthcare consultation in the past 12 months	43.3	
Any healthcare consultation in the past 3 months	40.6	
Non-specialist provider (i.e., General Practitioner, Family Medicine)	85.7	3.0 (1.9)
Psychiatrist	66.0	2.5 (1.7)
Psychologist	66.0	2.3 (1.6)
Use of medication		
Currently taking any prescription medications	28.0	
Frequency of consumption		
As needed	75.6	
Daily	24.4	
Duration of consumption		
Less than 1 month	20.7	
1 to 5 months	51.9	
6 months or more	27.4	
Serious medical events		
Any emergency department (ED) or hospitalization in the past 12 months	24.4	
ED visit only	15.1	2.1 (1.2)
ED visit with hospitalization	8.7	3.2 (2.5)
Direct hospitalization without ED visit	15.7	2.5 (1.8)

This table summarizes the healthcare utilization of main respondents who completed the longer survey on healthcare utilization and labor productivity (*N* = 483). Inclusion required a score of ≥3 on either the anxiety or depression subscale of the PHQ-4, passing the attention check, and completing the full survey. Questions on healthcare consultation in the past 3 months were only asked to respondents who reported that they had seen a healthcare provider in the last 12 months. Mean healthcare visits include tele and in person visits. Standard deviations provided in parentheses for continuous variables.

### 3.4 Labor productivity losses

Labor productivity costs are calculated based on the sample of employed respondents (*N* = 368). Employed respondents reported missing 272.5 h of work annually. Assuming an 8-h workday, this translates to missing an average of 34 days of work per person annually due to symptoms of anxiety and/or depression. Respondents reported an average presenteeism score of 5.1 (SD = 3.0) which means that they were 51 percent less productive while at work. This corresponds to 580 h of reduced productivity annually, or an average of 72.5 days per person lost to presenteeism. In total, symptoms of anxiety and depression contributed to more than 3 months of missed work per person each year.

### 3.5 Economic burden of anxiety and depression

Table 5 presents the breakdown of annual per person and total costs by direct and indirect costs and the overall economic cost of anxiety and depression among adults in Indonesia. Direct mental healthcare costs due to symptoms of anxiety and depression averaged IDR 2,111,020 (USD$ 133.99; 1 IDR = 0.000063 USD) per person. These per person costs are multiplied by the prevalence rate of 14.7 and population counts to obtain overall estimated healthcare costs of IDR 53,471.33 billion (USD$ 3.37 billion). Absenteeism and presenteeism costs due to symptoms of anxiety and depression averaged IDR 5,178,312 (USD$ 326.23) and IDR 11,021,700 (USD$ 694.37) per person, respectively. Labor productivity costs are extrapolated following the same approach for healthcare costs resulting in a total cost of IDR 410,340 billion (USD$ 25.85 billion). Summing up healthcare and labor productivity costs yields a total economic burden of anxiety and depression of IDR 463,811.33 billion (USD $29.2 billion). Absenteeism and presenteeism accounts for 88.5 percent of this total and healthcare costs account for the remaining 11.5 percent.

**Table 5:** Annual per person and total costs of depression and anxiety symptoms among adults in Indonesia.

Cost category	Per person costs (IDR & USD)	Total costs (IDR & USD BIL)	Share of costs (%)
Healthcare	IDR 2,111,020.00 USD$ 132.99	IDR 53,471.33USD$ 3.37	11.5
Absenteeism	IDR 5,178,312.00 USD$ 326.23	IDR 131,165.00 USD$ 8.26	28.3
Presenteeism	IDR 11, 021,700.00 USD$ 694.37	IDR 279,175.00 USD$ 17.55	60.2
Total	**IDR 18,311,032.00** **USD$ 1153.60**	**IDR 463,811.33** **USD $29.22**	**100.00**

This table summarizes the annual per person and total costs of depression and anxiety symptoms among adults in Indonesia. Per person costs are based on findings from main respondents who completed the longer survey on healthcare utilization and labor productivity (*N* = 483). Inclusion required a score of ≥3 on either the anxiety or depression subscale of the PHQ-4, passing the attention check, and completing the full survey. IDR denotes Indonesian Rupiah and USD denotes US dollars. Total costs are reported in billions.

In Supplementary material 2, we assess the robustness of these findings by comparing estimates with and without winsorization of continuous healthcare and labor productivity variables. Total costs were estimated at 2.3 percent of Indonesia’s GDP (IDR 494,999.82 billion/USD 31.19 billion), and applying winsorization to reduce the influence of extreme values lowered the estimate only slightly to 2.1 percent. This suggests that the results are robust and not overly sensitive to outliers.

### 3.6 Distribution of per capita productivity costs by severity of symptoms

To assess the distribution of per capita labor market costs by severity, we classify respondents based on the PHQ-9 and GAD-7 thresholds for minimal-mild, moderate, and moderately severe–severe symptoms. We also combine absenteeism and presenteeism costs. Over 60 per cent of respondents fall within the minimal to moderate categories. In general, there is a dose–response relationship where costs increase as symptom severity increases. Individuals experiencing moderate to severe depression symptoms report labor productivity costs that are IDR 7.8 million higher than those with minimal to moderate symptoms. Those with moderate symptoms report costs that are IDR 4.9 million higher compared to individuals with minimal to moderate symptoms. Individuals experiencing severe anxiety symptoms report IDR 7.7 million in higher costs compared to individuals with minimal to mild symptoms. The differences in costs between those in the minimal-mild vs. moderate categories are relatively small for depression and not significantly different for anxiety. Therefore, while severe symptoms incur the largest productivity costs, the higher prevalence of the milder categories and adverse effects on productivity also contribute to significant costs in the labor market. No significant differences are observed in healthcare expenditures by severity (Table 6).

**Table 6:** Distribution of per capita costs by severity of symptoms of main respondents in the sample with anxiety and/or depression.

Symptom severity	Proportion (%)	Per capita productivity costs (IDR MIL)	Test of difference
Depression severity			
Minimal-Mild (0–9)	37.3	12.1 (13.8)	Base
Moderate (10–14)	27.7	17.0 (15.1)	4.921 (1.908)*
Moderately Severe (>14)	35.1	19.9 (15.0)	7.806 (1.790)***
Anxiety severity			
Minimal-Mild (0–9)	48.6	13.7 (14.0)	Base
Moderate (10–14)	28.8	16.4 (15.1)	2.657 (1.797)
Severe (>14)	22.6	21.4 (15.4)	7.664 (1.947)***

This table summarizes the distribution of labor productivity costs by severity of anxiety/depression symptoms. Results are based on main respondents who completed the longer survey on healthcare utilization and labor productivity (*N* = 483). Inclusion required a score of ≥3 on either the anxiety or depression subscale of the PHQ-4, passing the attention check, and completing the full survey. Column 3 presents differences compared to the base group with standard errors provided in parentheses. *, **, ***Indicates significance at the 5, 1 and 0.1 percent levels, respectively. IDR denotes Indonesian Rupiah and costs are reported in millions.

## 4 Discussion

This is the first study to document the economic burden of anxiety and depression symptoms among adults in Indonesia. Healthcare costs averaged IDR 2,111,020 (USD$ 132.99) per person with these conditions. Indirect costs due to absenteeism and presenteeism averaged IDR 5,178,312 (USD$ 326.23) and IDR 11,021,700 (USD$ 694.37) per person. Based on a prevalence rate of 14.7 percent in the sample extrapolated to the population level, anxiety and depression among adults cost the Indonesian economy IDR 463,811.33 billion (USD $29.22 billion) per year. Healthcare costs account for 11.5 percent while declines in labor productivity accounts for over 80 percent of this cost. The overall economic burden accounts for 2.1 percent of Indonesia’s GDP as of 2023 (1.37 trillion USD$) (33). A similar study from Singapore found that these symptoms accounted for 2.9 percent of GDP (17). Prior studies from the US estimate the economic burden of diagnosed depression, excluding anxiety, to be 1.6 percent of GDP (4). Estimates of the costs of all mental health conditions, of which depression and anxiety account for the majority, are approximately 4 percent of GDP in Europe and the UK (5–7). The 2.1 percent estimate of GDP losses in Indonesia associated with anxiety and depression is within the range of these estimates. To put things in perspective, Indonesia spends approximately 3 percent of its GDP on healthcare expenditures with less than 2 percent of the national healthcare budget on mental health (34, 35). Therefore, as a proportion of Indonesia’s GDP, the economic burden of anxiety and depression represents more than half of the country’s total healthcare expenditures.

In our study, we showed that 14.7 percent of adults reported symptoms of anxiety and depression. This prevalence is higher than the prevalence rate of depression documented in the 2018 Basic Health Survey (6.1 percent) and lower than rates based on the most recent 2014–2015 IFLS wave (21.8 to 27.9 percent) (12, 14, 15). These differences in prevalence rates may arise from the use of different instruments. The Basic Health Survey used the Mini International Neuropsychiatric Interview (MINI) and the IFLS used the Centers for Epidemiologic Studies Depression Scale (CES-D-10) (12, 14). The former is a structured diagnostic interview, while the CES-D and PHQ-4 are self-reported tools, so the CES-D and PHQ-4 are likely to identify a higher number of probable cases. The differences in the depression prevalence rate we observe compared to the CES-D in the IFLS may be explained by the fact that our sample is more advantaged in terms of geographical distribution, educational attainment, and income compared to the general Indonesian population, making it less likely for them to experience mental health issues.

Over 60 percent of those with anxiety and depression symptoms in our sample had not been formally diagnosed by a healthcare provider. This suggests a high treatment gap and is comparable to estimates documented in other LMICs (10). Our diagnosis gap is larger than the treatment gap (90.7 percent) for depression documented in a recent study based on data from the 2018 Basic Health Survey (36). This is plausible given that our online sample is more educated and earns higher incomes, likely contributing to a greater awareness of mental health issues, help-seeking behaviors, and resources to access treatment. Our survey was administered online whereas the 2018 Basic Health was administered face to face by an interviewer. Prior studies show that under-reporting for potentially sensitive information may be more of a problem among interviewer administered as opposed to self-administered surveys (37). While the PHQ-4 has relatively high sensitivity and specificity in detecting diagnosed cases, it is important to note that not everyone who screens positive for symptoms will receive a clinical diagnosis of anxiety or depression. Our findings also reveal that the productivity burden of anxiety and depression is highest among individuals with severe symptoms. Nonetheless, respondents with mild and moderate symptoms also incur significant costs, underscoring the importance of early interventions. Addressing milder symptoms and preventing progression to more severe health states may mitigate overall costs. We did not find any significant differences in healthcare utilization by severity level which may reflect wider variation in individual-level preferences in help-seeking behavior.

This study estimates the economic burden of anxiety and depression using an inexpensive and expeditious approach. However, there are significant limitations. The main limitation is the reliance on an online panel. Compared to the general population, this sample is more advantaged in terms of income and educational attainment and thus may have greater awareness of and access to mental healthcare. Thus, we cannot guarantee that our sample with depression and anxiety symptoms is representative of the broader population with these symptoms. Moreover, while the PHQ-4 enables rapid identification of likely clinical symptoms of depression and anxiety, it is not an official diagnostic tool. While it has been validated internationally and among diabetes patients in Indonesia, it has not been validated for use yet in the general Indonesian population (38). Main respondents also filled out the PHQ-4 on behalf of other adult household members likely introducing reporting bias – it is difficult to predict the direction of this bias. Given the online nature of the survey, we were not able to conduct validation checks to assess the accuracy of these proxy reports. Further, there is a possibility of selection bias where individuals with greater levels of distress and/or mental healthcare use were more likely to respond to the full survey. We also excluded additional types of healthcare utilization, including over-the-counter medications, medical diagnostic tests, and services from faith or spiritual healers. Moreover, in our labor productivity costs calculations, we excluded fringe benefits as these tend to vary widely by employer and industry type and costs incurred due to unemployment linked to symptoms of anxiety and depression. Future studies should aim to corroborate these results using alternative methods, potentially relying on more representative samples and merging data with official medical records to obtain objective measures of healthcare utilization.

Since the majority of costs are incurred in the labor market, workplace interventions targeting employees may yield significant health and economic benefits. There is moderate evidence that workplace-focused mental health interventions can alleviate symptoms, although outcomes vary depending on the type and content of the intervention (39–41). Digital health interventions have shown promise due to their lower costs and the ability to be accessed privately through apps or other online platforms, which can help reduce stigma concerns (42, 43). A recent systematic review found that tailored digital interventions can effectively reduce anxiety and depression in employees experiencing high psychological distress, with positive effects on presenteeism, stress levels, sleep, and physical symptoms related to somatization among employees more broadly (44). In particular, workplace-based digital mental health interventions funded by the employer — combining lower-intensity, low-cost interventions (e.g., self-help tools) for mild symptoms with higher-intensity, higher-cost interventions (e.g., coaching or psychotherapy) for more severe symptoms — have shown promise in reducing symptoms and improving general wellbeing in Asian countries, while also minimizing the burden on the public healthcare system (45–48). Further research is needed to identify the most effective and cost-effective interventions in Indonesia’s context.

## 5 Conclusion

In conclusion, this study estimates that anxiety and depression among adults cost the Indonesian economy IDR 463,811.33 billion (USD $29.22 billion) per year, equivalent to 2.1 percent of the country’s GDP in 2023 (33). While the reliance on an online panel, use of self-reported screening tools, and omission of some cost categories present limitations, the results highlight the substantial burden of anxiety and depression. Given that the majority costs are incurred in the labor market, workplace-focused interventions hold considerable promise for reducing both symptoms and productivity losses. Evidence suggests that digital health tools, particularly when funded or facilitated by employers, can potentially offer scalable and cost-effective support. Further research is needed to adapt and evaluate these interventions in the Indonesian context to ensure effectiveness, affordability, and sustainability.

## Data Availability

The raw data supporting the conclusions of this article will be made available by the authors, without undue reservation.
