# Supplementary material for: The economic burden of anxiety and depression in Indonesia: evidence from a cross-sectional web panel survey
Source: Front Public Health. 2025 Oct 15;13:1667726. doi: 10.3389/fpubh.2025.1667726 (PMC12570936; doi:10.3389/fpubh.2025.1667726)
Supplement: Supplementary file 1 [file Supplementary_file_1.docx]

**Supplementary Materials**

**Supplementary Material 1**

The unit costs below were sourced from the Regulation of the Ministry of Health in Indonesia (Permenkes Number 3/ 2023). Costs used in the analyses are bolded. For our calculations, we use a duration of 2 weeks, 3 months, and 8 months for medications taken less than a month, one to six months, and for more than six months respectively.

| Type | Average unit costs (IDR) | Unit costs used in analyses (IDR) |
| --- | --- | --- |
| Medication |  |  |
| Benchmark – Daily for a month | 50,000-100,000 | 75,000 |
| Daily for less than one month | 25,000 | 37,500 |
| Daily for one to six months | 150,000 | 225,000 |
| Daily for more than six months | 400,000 | 600,000 |
| As needed for less than one month | 25,000 | 18,750 |
| As needed for one to six months | 75,000 | 112,500 |
| As needed for more than six months | 200,000 | 300,000 |
| Non-specialist consultation | 3,600 – 16,000 | 9,800 |
| Psychiatrist consultation | 30,000 – 50,000 | 40,000 |
| Psychologist consultation | 30,000 – 50,000 | 40,000 |
| Emergency department visit | 180,000 | 180,000 |
| Emergency department visit and hospital admission | 4,414,000 – 11,676,000 | 8,045,000 |
| Hospital admission (per night) | 4,234,000 – 11,496,000 | 7,865,000 |

**Supplementary Material 2**

On average, individuals reported 38 missed workdays due to symptoms of anxiety and depression. They also reported being 51 percent less productive while at work, which is equivalent to an additional 76 lost workdays attributable to presenteeism.

The table below presents the breakdown of annual per person and total costs by direct and indirect costs and the overall economic cost of anxiety and depression among adults in Indonesia without winsorization on the continuous variables. Direct mental healthcare costs due to symptoms of anxiety and depression averaged IDR 2,119,013 (USD$ 133.50; 1 IDR = 0.000063 USD) per person. These per person costs are multiplied by the prevalence rate of 14.7 and population counts to obtain overall estimated healthcare costs of IDR 53,673.79 billion (USD$ 3.38 billion). Absenteeism and presenteeism costs due to symptoms of anxiety and depression averaged IDR 5,827,770 (USD$ 367.15) and IDR 11,595,550 (USD$ 730.52) per person respectively. Labour productivity costs are extrapolated following the same approach for healthcare costs resulting in a total cost of IDR 441,326 billion (USD$ 27.80 billion). Summing up healthcare and labour productivity costs yields a total economic burden of anxiety and depression of IDR 494,999.82 billion (USD $31.19 billion). Absenteeism and presenteeism accounts for 89.1 percent of this total and healthcare costs account for the remaining 10.8 percent. The total costs represent 2.3 percent of Indonesia’s GDP compared to 2.1 percent when winsorization at the 95^th^ percentile is applied.

Annual per person and total costs of depression and anxiety symptoms among adults in Indonesia (without winsorization)

| Cost category | Per person costs (IDR & USD) | Total costs (IDR & USD BIL) | Share of costs (%) |
| --- | --- | --- | --- |
|  |  |  |  |
| Healthcare | IDR 2,119,013.00  USD$ 133.50 | IDR 53,673.79  USD$ 3.38 | 10.8 |
| Absenteeism | IDR 5,827,770.00  USD$ 367.15 | IDR 147,615.18  USD$ 9.30 | 29.8 |
| Presenteeism | IDR 11, 595,550.00  USD$ 730.52 | IDR 293,710.85  USD$ 18.50 | 59.3 |
| **Total** | **IDR 19,542,333**  **USD$ 1,231.17** | **IDR 494,999.82**  **USD $31.19** | **100.00** |

Notes: This table summarizes the annual per person and total costs of depression and anxiety symptoms among adults in Indonesia. Per person costs are based on findings from main respondents who completed the longer survey on healthcare utilization and labour productivity (N=483). Inclusion required a score of ≥3 on either the anxiety or depression subscale of the PHQ-4, passing the attention check, and completing the full survey. IDR denotes Indonesian Rupiah and USD denotes US dollars. Total costs are reported in billions.
